# New Appliances and Things Medical

**Published:** 1895-02-23

**Authors:** 


### NEW APPLIANCES AND THINGS MEDICAL.

[We shall be glad to receive, at our Office, 428, Strand, London, W.C., from the manufacturers, specimens of all new preparations and appliances which may be brought out from time to time.]

### MARROL.

(The Liquor Carnis Company, 28a, Farringdon Street, London, E.C.)

The Liquor Carnis Company are already well known as food specialists, and as such have the experience and knowledge requisite for the preparation of satisfactory scientific foods. In Marrol they have succeeded in combining the yellow marrow of ox bone with hopped extract of malt in physiological proportions. It is well known that red bone marrow is a fat which is very readily assimilated, and as it contains an appreciable quantity of hæmoglobin and organic compounds containing iron and phosphorus, it has recently found favour in the treatment of anæmia. Marrol resembles an emulsion of malt extract and cod liver oil in appearance, but is most agreeable to the taste, and should be acceptable to those persons who cannot take cod liver oil. From our analysis of this preparation we find that the amount of marrow fat present is considerable, and thus makes it a valuable fattening food. We have further detected a large amount of calcium phosphate in this preparation, which should make it of value as a food for growing children. The amount of maltose present makes the preparation sweet to the taste, but this flavour is somewhat masked by the hops which have been judiciously added. In addition to these ingredients we find indications of albuminoid matter, which considerably increases the nutritive value of the preparation. In the samples we have examined we found the diastasic action of the malt to be small, and are pleased to find that no preservatives have been added which might have any deleterious influence upon those who may be inclined to take this food stuff regularly.

### PERIODATE COMPOUNDS.

We have received from the Periodate Compounds Company, Manor Street Works, Clapham, samples of their preparations, viz., a solution of Hydrarg. Periodate, and the same drug in the form of a strong and a mild ointment. Briefly stated, it is claimed that mercury in this form is less poisonous and practically as active an antiseptic as in the more familiar perchloride form. The preparations are well made, and pleasant to use. We have tried the ointments, and find them comfortable to the patient, and antiseptic in their action. The liquid has also yielded good results as an antiseptic in our hands.

